# Temperature Effects on Wicking Dynamics: Experimental and Numerical Study on Micropillar-Structured Surfaces

**DOI:** 10.3390/mi16050512

**Published:** 2025-04-27

**Authors:** Yoomyeong Lee, Hyunmuk Park, Hyeon Taek Nam, Yong-Hyeon Kim, Jae-Hwan Ahn, Donghwi Lee

**Affiliations:** 1 Graduate School of Mechanical-Aerospace-Electric Convergence Engineering, Jeonbuk National University, 567 Baekje-Daero, Deokjin-gu, Jeonju-si 54896, Jeollabuk-do, Republic of Korea; lkwon24@jbnu.ac.kr (Y.L.); qwq4943@jbnu.ac.kr (H.P.); skagusxor@jbnu.ac.kr (H.T.N.); 2 Department of Mechanical Engineering, Yonsei University, 50 Yonsei-ro, Seodaemun-gu, Seoul 03722, Republic of Korea; yhkim0054@yonsei.ac.kr; 3 Food Safety and Distribution Research Group, Korea Food Research Institute, Wanju-gun 55365, Jeollabuk-do, Republic of Korea; jhahn@kfri.re.kr; 4 Department of Mechanical System Engineering, Jeonbuk National University, 567 Baekje-Daero, Deokjin-gu, Jeonju-si 54896, Jeollabuk-do, Republic of Korea; 5 Advanced Transportation Machinery Research Center, Jeonbuk National University, 567 Baekje-Daero, Deokjin-gu, Jeonju-si 54896, Jeollabuk-do, Republic of Korea

**Keywords:** critical heat flux, wicking performance, micropillar-structured surface, computational fluid dynamics, infrared visualization

## Abstract

Boiling heat transfer, utilizing latent heat during phase change, has widely been used due to its high thermal efficiency and plays an important role in existing and next-generation cooling technologies. The most critical parameter in boiling heat transfer is critical heat flux (CHF), which represents the maximum heat flux a heated surface can sustain during boiling. CHF is primarily influenced by the wicking performance, which governs liquid supply to the surface. This study experimentally and numerically analyzed the wicking performance of micropillar structures at various temperatures (20–95 °C) using distilled water as the working fluid to provide fundamental data for CHF prediction. Infrared (IR) visualization was used to extract the wicking coefficient, and the experimental data were compared with computational fluid dynamics (CFD) simulations for validation. At room temperature (20 °C), the wicking coefficient increased with larger pillar diameters (D) and smaller gaps (G). Specifically, the highest roughness factor sample (D04G10, *r* = 2.51) exhibited a 117% higher wicking coefficient than the lowest roughness factor sample (D04G20, *r* = 1.51), attributed to enhanced capillary pressure and improved liquid supply. Additionally, for the same surface roughness factor, the wicking coefficient increased with temperature, showing a 49% rise at 95 °C compared to 20 °C due to reduced viscous resistance. CFD simulations showed strong agreement with experiments, with error within ±10%. These results confirm that the proposed numerical methodology is a reliable tool for predicting wicking performance near boiling temperatures.

## 1. Introduction

Boiling heat transfer is a highly efficient thermal transport mechanism that utilizes the large latent heat released during phase change from liquid to vapor. It has been widely applied in advanced thermal management systems such as nuclear reactor core cooling, data center cooling, and semiconductor chip cooling, and is still considered important for both current and next-generation cooling technologies for electronic devices [1,2,3,4,5,6,7,8]. The most critical parameter in boiling heat transfer is the critical heat flux (CHF), which represents the maximum heat flux a heated surface can endure during the boiling process. When CHF is reached, a thin vapor film forms on the surface, preventing liquid supply and drastically reducing heat transfer efficiency. Since the thermal conductivity of the vapor film is significantly lower than that of the liquid phase, it acts as an insulator, leading to rapid temperature rise and potential surface failure. Therefore, enhancing CHF is essential for maintaining stable and efficient heat transfer. To improve CHF, sufficient liquid supply to the heated surface must be ensured, which is quantified by the wicking performance. Wicking refers to the amount of liquid transported to the surface by capillary forces. Higher wicking performance enhances liquid replenishment during boiling and promotes bubble departure, ultimately improving CHF [9,10,11,12]. One of the most effective methods for enhancing wicking performance is surface modification using micro/nanostructures [13,14]. Porous microstructures generate strong capillary forces, facilitating liquid supply. Consequently, many researchers have focused on optimizing the pore size of micro/nanostructures to enhance liquid transport efficiency. Previous studies have demonstrated that microstructures generally outperform nanostructures in liquid transport capability. In particular, micropillar structures have been shown to form multiple fluid flow channels, significantly improving liquid supply efficiency [9]. As a result, extensive research has been conducted to evaluate the wicking performance of micropillar structures and improve CHF prediction models accordingly [15,16].

One of the important criteria for assessing the wicking conditions of a surface is the critical contact angle, which is defined by Equation (1):

(1) $$\theta_{c}=\cos^{-1}\frac{1-\varphi }{r-\varphi }$$

Here, r and φ represent the roughness factor and the solid fraction, respectively. The roughness factor, first introduced by Wenzel [17], is used to describe the wettability on roughened surfaces. It is defined by Equation (2):

(2) $$r=\frac{A_{actual}}{A_{projection}}=\frac{{\left(D+G\right)}^{2}+\frac{\pi }{2}\cdot \pi DH}{{\left(D+G\right)}^{2}}$$

$A_{actual}$ includes a coefficient of π/2 to account for the scalloped sidewalls of the micropillars, which result from the deep reactive ion etching (DRIE) process [18]. It is known that wicking occurs when the apparent contact angle (θ) is smaller than the critical contact angle ($\theta_{c}$). Wicking experiments are essential for establishing a CHF correlation, as they allow for the quantitative evaluation of liquid replenishment to the heated surface during bubble departure in the boiling process. Therefore, numerous studies have measured and analyzed wicking performance to improve CHF prediction models [9,10,11,12,15,16]. However, previous studies have mainly developed CHF prediction models based on wicking coefficients measured at room temperature (20 °C), which limits their accuracy. Since liquid supply behavior near the actual boiling temperature (~100 °C) is not well reflected, the accuracy of these models is reduced.

**Figure 1:**
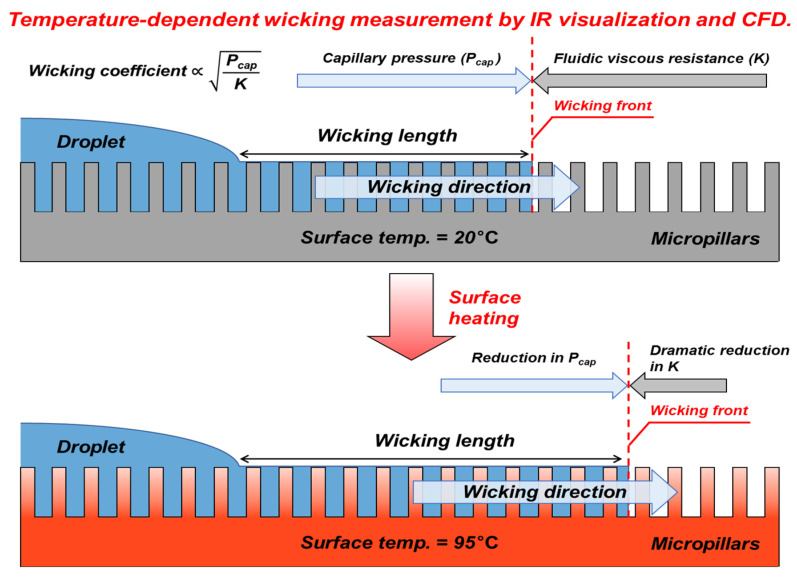
A schematic of wicking performance under temperature variation.

To address this limitation, this study aims to analyze the effect of temperature on wicking performance using experiments and CFD simulations, as illustrated in Figure 1, and to establish a more precise CHF prediction model. Although initial attempts were made to conduct experiments at 100 °C under saturated humidity conditions, the droplets evaporated too rapidly, posing experimental limitations. Therefore, 95 °C was selected as the maximum experimental temperature, as it minimizes the variation in the key properties of the working fluid (distilled water), effectively suppresses rapid droplet evaporation, and closely resembles actual boiling conditions. Based on this, wicking coefficients were measured and simulated in various temperature ranges (20–95 °C), providing a database for developing a new CHF correlation. For this purpose, four types of micropillar samples with different diameters and gaps were fabricated, and their wicking performance was experimentally evaluated. Additionally, an infrared (IR) visualization technique was applied to accurately measure wicking behavior, and Fluent-based CFD simulations were conducted to compare with experimental results and validate the reliability of the numerical approach.

## 2. Research Methods

### 2.1. Experimental Methods

#### 2.1.1. Micropillar Sample Fabrication

In this study, micropillar samples for wicking tests were fabricated using the MEMS process. The MEMS process is a representative manufacturing technique that enables precise patterning of microstructures, thereby enhancing the uniformity and geometric accuracy of micropillar structures. The substrates used for fabrication were silicon wafers (double-sided polished, 100, 1~10 Ω), and a chemical cleaning process was conducted to remove surface impurities and ensure uniform etching. First, organic contaminants were removed using a piranha solution (H_2_O_2_/H_2_SO_4_, volume ratio 1:3), followed by the removal of the oxide layer using a buffered oxide etchant (BOE, HF/H_2_O, volume ratio 1:5). This pretreatment process ensured a clean wafer surface, enabling uniform pattern formation in subsequent photolithography and etching steps. After the pretreatment, a photoresist (PR) was uniformly coated on the silicon wafer surface, and a micro-dot array pattern was formed through UV lithography and development. The desired pillar shape was patterned in a dot form using a mask pattern, and unnecessary PR was removed through the development process. The micropillar structures were then formed using the DRIE process. The DRIE process was conducted using the Bosch process, where SF_6_ and C_4_F_8_ gases were alternately introduced to achieve etching and protection steps, leading to the fabrication of high-aspect-ratio micropillars. During the etching process, a polymer protective layer (C_4_F_8_) was deposited to prevent excessive etching of the pillar sidewalls. Finally, an asher process was performed to remove the PR and protective layer on top of the pillars, completing the micropillar structures. The fabricated micropillar structures were categorized based on their diameter (D) and gap (G), designed with diameters of 4, 10, and 20 μm and gaps of 10 and 20 μm. Detailed specifications of the samples used in the experiments are provided in Table 1.

**Figure 2:**
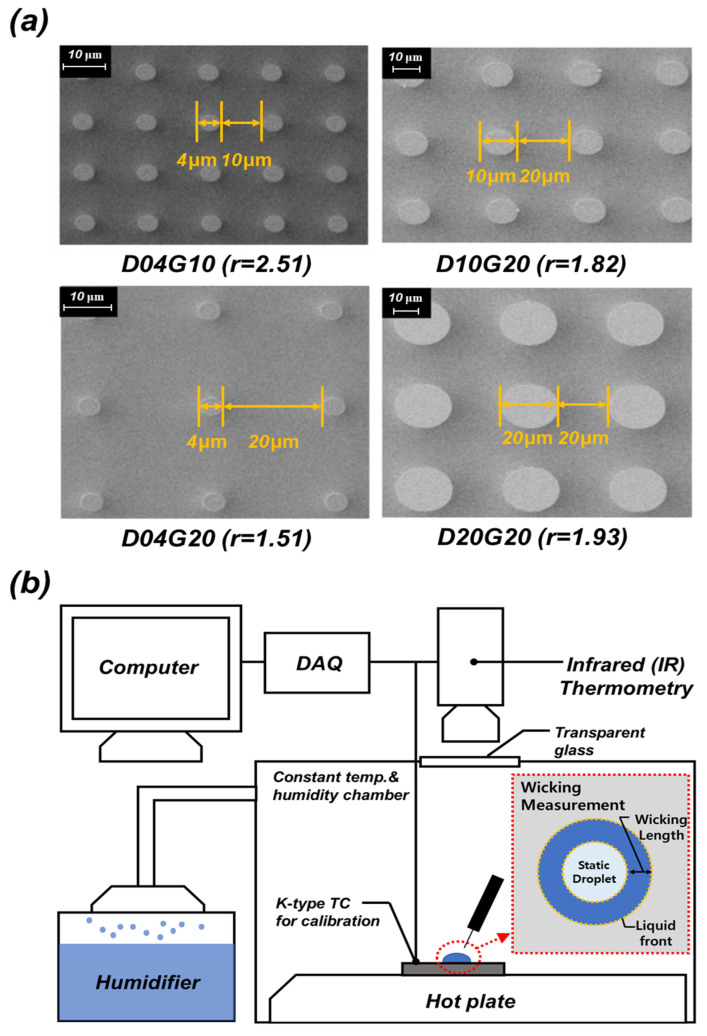
(**a**) SEM Images of micropillar samples; (**b**) Schematic diagram of the experimental setup for wicking performance measurement.

Finally, scanning electron microscopy (SEM) was used to evaluate the morphology and surface uniformity of the micropillars, and the SEM images are shown in Figure 2a. Further details on the fabrication process and process parameters are described in references [19,20].

#### 2.1.2. Wicking Measurement

We conducted wicking experiments to evaluate the fluid supply performance of the fabricated micropillar samples. Figure 2b shows a schematic diagram of the experimental setup used for wicking measurements. The test samples were mounted on a hot plate (MS-300HS, Misung Scientific Instruments Co., Ltd., Yangju, Republic of Korea), and the surface temperature was controlled within a range of 20 °C to 95 °C during the experiments. A micropipette was used to deposit a 15 μL droplet onto the test samples. As the surface temperature increased, evaporation of the droplet was accelerated; therefore, a humidifier was used to maintain a saturated humidity environment and suppress evaporation. The wicking test apparatus was placed inside an acrylic chamber, and all tests were conducted under controlled humidity and temperature conditions. When the droplet came into contact with the test sample, a liquid boundary was formed, and the wicking phenomenon caused the liquid to spread isotropically. The moving fluid boundary was defined as the wicking front, and the wicking length (i.e., the distance between the wicking front and the droplet boundary) was quantitatively measured over time. In general, as the droplet spread due to wicking, the static boundary of the droplet could change slightly. However, this was a natural occurrence in the liquid movement process on the micropillar sample and did not affect the wicking measurement. To quantitatively analyze the wicking phenomenon, an IR camera (FLIR A400, Teledyne FLIR LLC, Wilsonville, OR, USA) and an LED lamp (SL-200W II, Godox Photo Equipment Co., Ltd., Shenzhen, China) were used to capture the wicking process.

#### 2.1.3. IR Visualization

To quantitatively analyze the wicking phenomenon, an IR visualization technique was utilized to measure the wicking length. The IR camera was positioned perpendicularly above the micropillar sample to capture the real-time progression of the wicking phenomenon after the droplet made contact with the surface. The recorded data underwent post-processing to precisely analyze the wicking length. The IR camera detected temperature signal differences caused by the emissivity contrast between the droplet and the silicon wafer, enabling clear visualization of the liquid movement boundaries. In this experiment, the IR visualization images were used to quantitatively measure the displacement of the wicking front over time, with the wicking length determined by analyzing the distance between the droplet boundary and the wicking front. The experimental setup was housed inside an acrylic chamber to maintain stable environmental conditions, and a humidifier was used for humidity control. The captured IR images were processed using ImageJ 1.53e software, extracting the movement path of the wicking front and converting it into quantitative data. This allowed for the evaluation of wicking length variations under different temperature conditions and an analysis of how surface temperature influences wicking performance.

The IR visualization technique applied in this study focused on accurately measuring wicking length rather than analyzing the thermal characteristics of the wicking phenomenon. This approach contributed to precisely quantifying the fluid transport behavior on the micropillar sample.

#### 2.1.4. Uncertainty

The uncertainty in the wicking experiments was evaluated at a 95% confidence level. The wicking length was defined as the distance between the static boundary and the dynamic wicking liquid front, and uncertainties could arise due to the resolution of captured images and the accuracy of measurement equipment. In this study, the IR camera used for data acquisition had a resolution of approximately 200 μm per pixel. Consequently, the measurement error due to image resolution was estimated to be ±1 pixel (±200 μm). Additionally, surface temperature measurements were conducted using a K-type thermocouple, and the uncertainty was determined to be ±1.1 °C, considering the accuracy of the measurement equipment and minor fluctuations in data acquisition. The temperature control error of the hot plate (MS-300HS) used in the experiment was evaluated to be less than 0.5 °C. Furthermore, a humidifier was employed to maintain consistent humidity levels, thereby minimizing additional uncertainties caused by environmental variations. The uncertainty in the wicking coefficient and volumetric wicking rate was assessed using Moffat’s uncertainty analysis method [21]. The wicking coefficient was calculated using Equation (3):

(3) $$W=\frac{l}{\sqrt{t}}$$

where l represents the wicking length, defined as the distance between the static boundary and the dynamic wicking liquid front. The analysis results indicated that the uncertainty in the wicking coefficient was approximately 4.5%, while the uncertainty in the wicking velocity was 6.4%. To minimize errors in wicking length measurement, experiments were repeated under identical conditions, and the final values were determined based on the average, considering the variability in the measured data.

### 2.2. Numerical Methods

#### 2.2.1. Physical Model and Boundary Conditions

**Figure 3:**
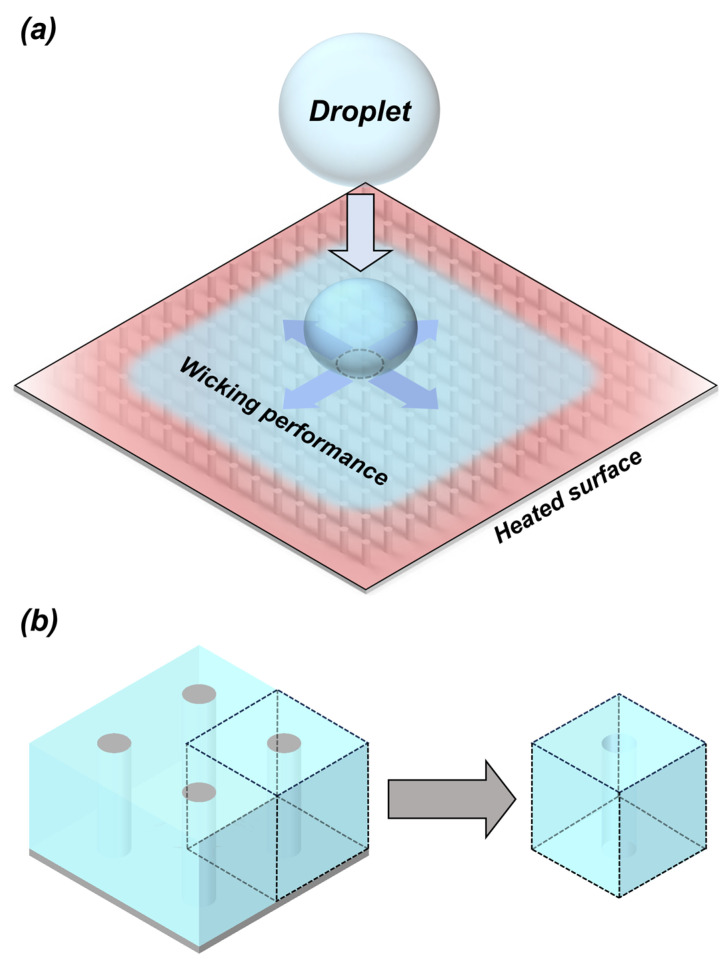
(**a**) A schematic diagram of wicking phenomenon; (**b**) the numerical domain used for the CFD simulation.

In this study, ANSYS Fluent 18.1 was utilized to numerically analyze the wicking phenomena in micropillar structures. Figure 3a presents a schematic of the wicking phenomena, while Figure 3b illustrates the numerical domain used for the CFD simulation. Simulating the entire micropillar structure would require a significant computational cost; therefore, a periodic unit cell approach was applied to model the overall wicking behavior efficiently. The micropillar samples used in the numerical analysis were classified into four cases based on pillar diameter (D) and pillar gap (G): D04G10, D04G20, D10G20, and D20G20. The pillar diameters (D) were set to 4, 10, and 20 μm, while the pillar gaps (G) were 10 and 20 μm. The thermophysical properties of water were obtained from the NIST chemistry database. Since the fluid flow in the micropillar structure exhibits a low Reynolds number, a laminar flow model was applied for the simulation. The computational grid was generated using the ICFD-CFD 18.1 commercial software, employing an O-grid method to create a high-quality grid. To enhance the accuracy of the numerical analysis, a hexahedral mesh was used, and the wall function was set to maintain y^+^ below 1, ensuring sufficient resolution of the viscous boundary layer. For fluid flow analysis, periodic boundary conditions were applied, with the inlet and outlet positioned at the front and back of the numerical domain. The pressure gradient was set to 50,000 Pa/m based on reference [22], ensuring that the mean velocity was proportional to the imposed pressure gradient, thereby maintaining a constant viscous resistance (*K*). The viscous resistance (*K*) was calculated using Equation (4):

(4) $$K=\frac{\left(dP/dx\right)}{u_{mean}}$$

dP/dx represents the pressure gradient, $u_{mean}$ denotes the average flow velocity through the micropillar structure. A no-shear stress condition was applied to the liquid-air interface (top boundary) to eliminate shear stress, while a no-slip condition was imposed on the solid-liquid interface (bottom and pillar surfaces) to set the velocity to zero at the walls. Additionally, symmetric boundary conditions were applied to the side boundaries to ensure symmetrical fluid flow. These boundary conditions were implemented to accurately replicate the actual fluid behavior within the micropillar structure.

#### 2.2.2. Grid Independence Test

**Figure 4:**
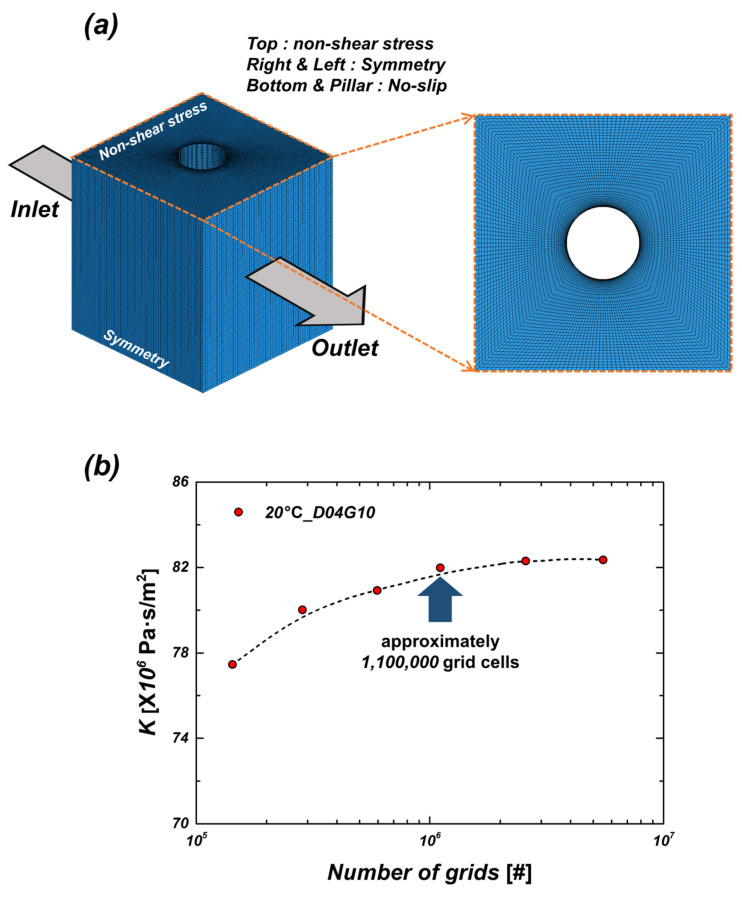
(**a**) Grid and boundary conditions for CFD simulation; (**b**) the grid independence test.

Figure 4a illustrates the grid and boundary conditions used in the simulation. To accurately capture the shear stress effects on the micropillar surface, a prism layer was generated around the pillar region, allowing precise representation of velocity variations and viscous resistance at the walls. Additionally, high-quality hexahedral meshes were employed to enhance the accuracy of the simulation. The grid independence test results are shown in Figure 4b. Therefore, selecting an optimal mesh size is essential for balancing computational efficiency and accuracy. The grid independence test was conducted at 20 °C under the D04G10 sample condition. A total of six different mesh densities (ranging from approximately 10^5^ to 10^7^ elements) were tested by comparing the viscous resistance values. The results showed that beyond approximately 1,100,000 elements, changes in viscous resistance values were negligible, indicating that further increasing the mesh density would not significantly improve accuracy. Consequently, a mesh size of approximately 1,100,000 elements was selected to ensure accurate simulations while minimizing computational cost.

#### 2.2.3. Model Validation

**Figure 5:**
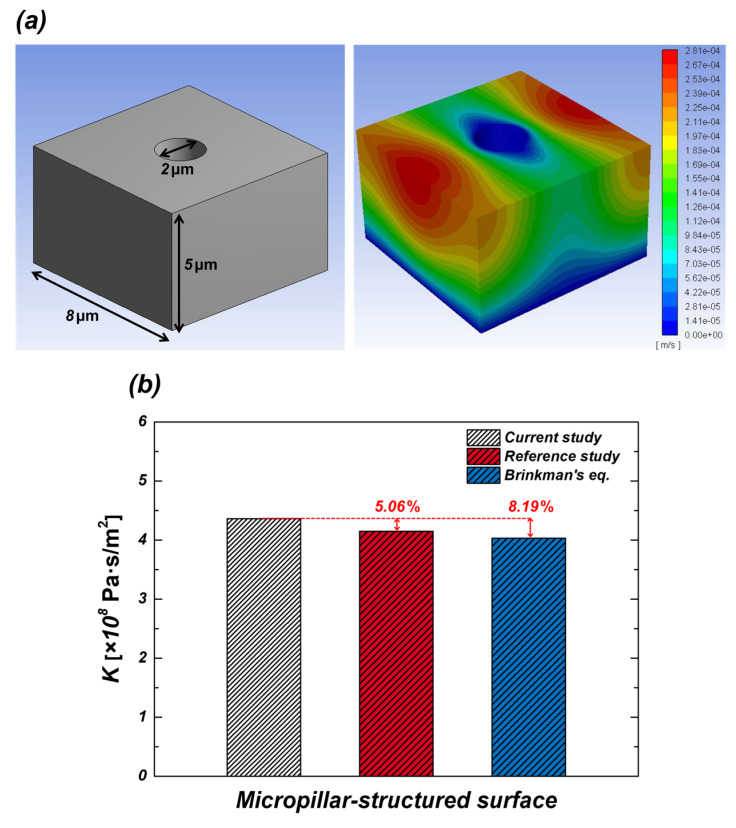
(**a**) The geometry used for validation; (**b**) comparison of viscous resistance results [22,23].

It is difficult to implement ideal experimental conditions due to several factors, such as droplet evaporation, anisotropic propagation during wicking, differences in image resolution, and data acquisition frequency. These uncertainties affect the experimental results and lead to discrepancies from numerical analysis. Therefore, comparison between experimental and simulation results is crucial to ensure the accuracy of the numerical model. To validate the numerical model, its results were compared with references [22,23]. The wicking behavior in micropillar structures was analyzed using ANSYS Fluent, with viscous resistance chosen as the primary evaluation metric. Figure 5 illustrates the geometry used for validation and comparison of viscous resistance results. The CFD-based viscous resistance proposed by Xiao et al. [22], and the theoretical viscous resistance, calculated using Brinkman’s equation [23], were each compared with the numerical results obtained in this study. The relative errors between the simulation result of this study and the reference value were 5.06% and 8.19%, respectively. These results demonstrate that the numerical methodology proposed in this study has been validated for its reliability in predicting the wicking behavior in micropillar structures, when compared with the numerical approaches proposed in the reference [22] and theoretical predictions [23].

## 3. Results

### 3.1. Wicking Analysis Under Temperature Variation Using IR Visualization

#### 3.1.1. IR-Based Wicking Analysis at 20 °C

The wicking performance of four different micropillar samples with varying diameters and pillar gaps was analyzed at room temperature (20 °C) using IR visualization. The wicking length on the micropillar surface is determined by the relationship between capillary pressure and viscous resistance, which can be expressed by the following Equation (5):

(5) $$l=\sqrt{\frac{2P_{cap}}{K}t}=W\sqrt{t}$$

**Figure 6:**
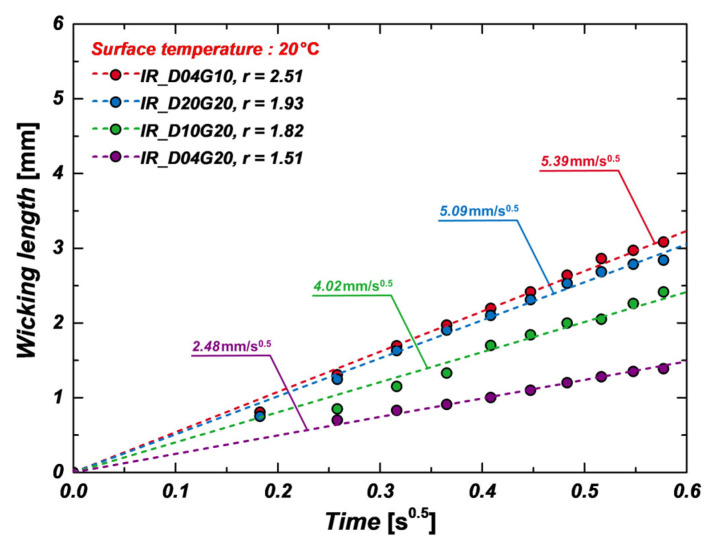
Wicking coefficients of micropillar samples at 20 °C measured using IR visualization.

Here, $P_{cap}$ represents capillary pressure. Capillary pressure serves as the driving force for liquid movement and increases when the pillar gap decreases or when the solid-liquid contact area per unit volume increases. Conversely, viscous resistance acts as a force that impedes liquid flow and decreases as the viscosity lowers. Figure 6 presents a comparative analysis of the wicking coefficients of micropillar samples measured at 20 °C using IR visualization, illustrating the impact of micropillar geometry on wicking performance. The wicking coefficient represents the rate of increase in wicking length, and the experimental results indicate that narrower pillar gap (G) leads to a higher wicking coefficient. The D04G10 sample (pillar gap of 10 μm) exhibited approximately 117% higher wicking performance compared to the D04G20 sample (pillar gap of 20 μm). This is attributed to the increased capillary pressure as the pillar gap decreases. Additionally, the wicking coefficient tended to increase with larger pillar diameters (D), as larger pillar diameters enhance the solid-liquid contact area per unit volume, generating stronger capillary forces. Specifically, the wicking coefficient of the D20G20 sample (diameter of 20 μm) was measured at approximately 5.09 mm/s^0.5^, which is 105% and 27% higher than that of the D04G20 (diameter of 4 μm) and D10G20 (diameter of 10 μm) samples, respectively. As previously mentioned, wicking performance can be improved by reducing pillar gap or increasing pillar diameter to enhance capillary pressure. These geometric factors can be integrated into the concept of surface roughness factor.

#### 3.1.2. IR-Based Wicking Analysis Under Temperature Increase

As previously mentioned, in the actual boiling heat transfer process, the fluid supply characteristics change as the heating surface temperature increases, directly affecting boiling heat transfer performance and CHF. Therefore, analyzing the wicking performance under varying temperature conditions is crucial for precisely understanding the fluid supply mechanism in boiling environments. To achieve this, the surface temperature was incrementally increased, and the wicking performance was quantitatively measured using IR visualization. The wicking experiments were conducted at surface temperatures of 20 °C, 55 °C, 75 °C, and 95 °C.

**Figure 7:**
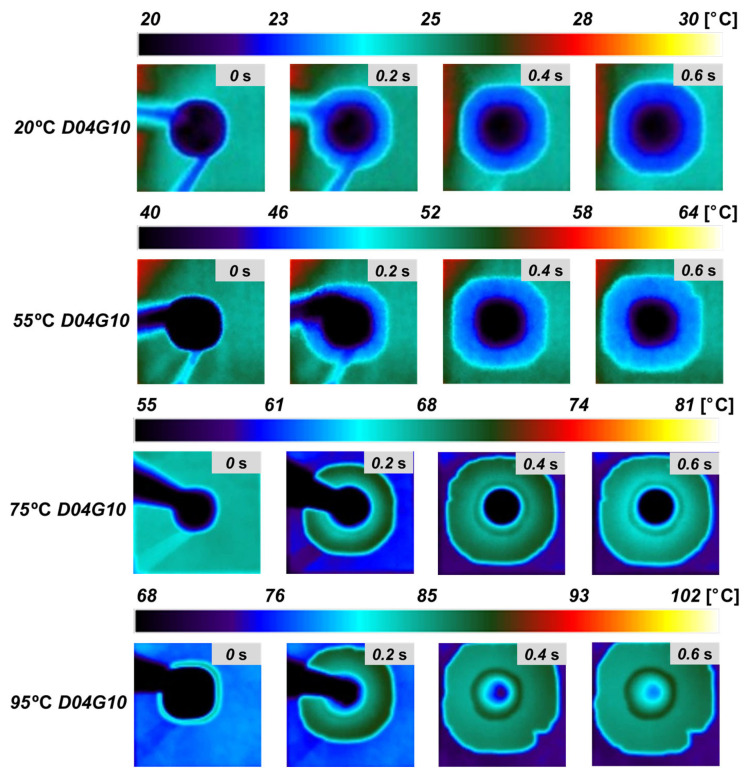
IR images of wicking length at various temperatures for the D04G10 sample.

**Figure 8:**
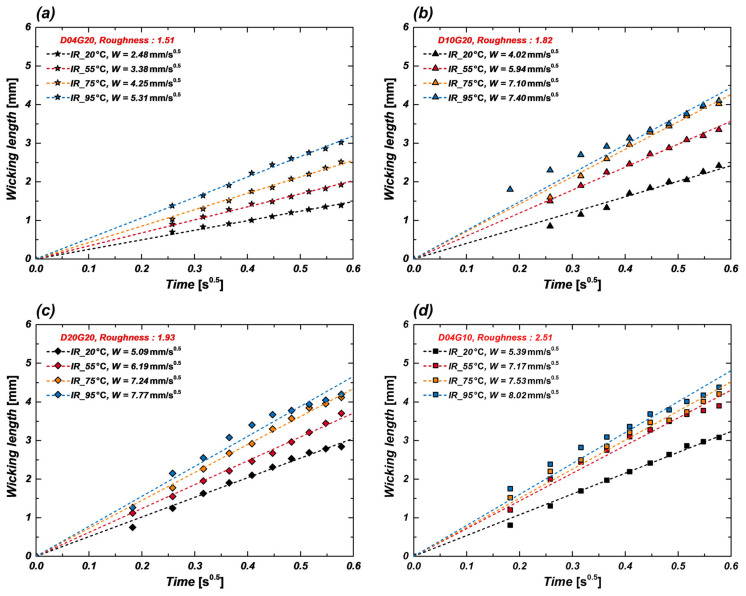
Wicking coefficients of four samples with increasing surface temperature: (**a**) D04G20; (**b**) D10G20; (**c**) D20G20; (**d**) D04G10.

Figure 7 presents IR images of wicking length at different temperatures for the D04G10 sample. These images clearly demonstrate that wicking length increases as surface temperature rises. This can be attributed to the significant reduction in flow resistance due to decreased fluid viscosity at higher temperatures, whereas the reduction in capillary pressure due to decreased surface tension is relatively smaller. Consequently, as represented by Equation (5), the wicking coefficient increases with temperature, indicating that fluid supply becomes more efficient. Additionally, the IR images enable clear identification of the boundary between the liquid (water) and solid (silicon wafer), allowing quantitative analysis of the wicking front’s movement. Figure 8a–d compare the wicking coefficients of four samples (D04G20, D10G20, D20G20, D04G10) at different temperatures (20 °C, 55 °C, 75 °C, and 95 °C). The experimental results show that samples with higher surface roughness factor exhibit greater wicking coefficients and that the wicking coefficient generally increases with temperature. Specifically, at 95 °C, the wicking coefficient increased by approximately 114% in D04G20 sample (*r* = 1.51), 84% in D10G20 sample (*r* = 1.82), 53% in D20G20 sample (*r* = 1.93), and 49% in D04G10 sample (*r* = 2.51) compared to their respective values at 20 °C. This trend is attributed to the strong capillary pressure generated by narrow pillar gap and high surface roughness factor. However, at high temperatures (95 °C), the rate of increase in the wicking coefficient was relatively lower. This suggests that additional physical factors at elevated temperatures may influence wicking performance beyond the simple effect of reduced viscous resistance. Measurement uncertainty in the wicking experiments may have contributed to this trend. Considering the resolution and frame rate of the IR camera, an uncertainty of approximately 4.5% exists in the wicking length measurement, which may weaken data linearity. Some samples exhibited anisotropic wicking behavior, where wicking progressed more rapidly in specific directions. This may be due to minor shape variations introduced during fabrication, differences in initial droplet contact conditions, or small surface non-uniformities. To minimize this error, wicking lengths were measured in four directions, and their average values were used in the analysis. Evaporation effects at high temperatures may also have affected the results. At temperatures above 80 °C, maintaining a fully saturated humidity environment is challenging, increasing the likelihood of droplet evaporation. This may have led to underestimation of the actual wicking length. A humidifier was used to maintain a sufficiently humid environment during experiments, but minor evaporation may still have occurred. Despite these factors, the correlation coefficient (R^2^) of the experimental data remained above 0.96, indicating that the slope of the fitted regression line still reliably represents the wicking coefficient. In this study, the wicking phenomenon was quantitatively measured using IR visualization, and a systematic analysis of wicking performance under different temperature conditions was conducted. The results confirm that wicking performance improves with increasing temperature in all samples, and the closer to the boiling conditions, the better fluid supply capability.

### 3.2. Wicking Analysis Under Temperature Variation Using CFD

IR visualization was used to evaluate wicking performance experimentally. However, to quantitatively analyze the effects of micropillar geometry and temperature on capillary pressure and viscous resistance, CFD simulations were performed as well.

The wicking coefficients obtained from CFD simulations were generally higher than those from IR-based experiments. This discrepancy is attributed to the idealized boundary conditions used in numerical models, such as perfect saturation and neglect of droplet evaporation, which resulted in lower viscous resistance in simulations compared to actual experiments. Additionally, while evaporation and thermal effects are present in IR visualization experiments, CFD simulations have limitations in fully accounting for these factors. To correct these differences, a fitting factor was applied between experimental and CFD data. By comparing CFD results with experiments, a fitting factor of 0.468 was determined to provide the highest accuracy. Applying this correction allowed the predicted wicking coefficients from CFD to match experimental values more closely, enabling a more reliable evaluation of wicking trends under varying microstructure and temperature conditions. Furthermore, numerical validation was conducted for four different samples (D04G10, D04G20, D10G20, D20G20).

#### 3.2.1. CFD-Based Wicking Analysis at 20 °C

**Figure 9:**
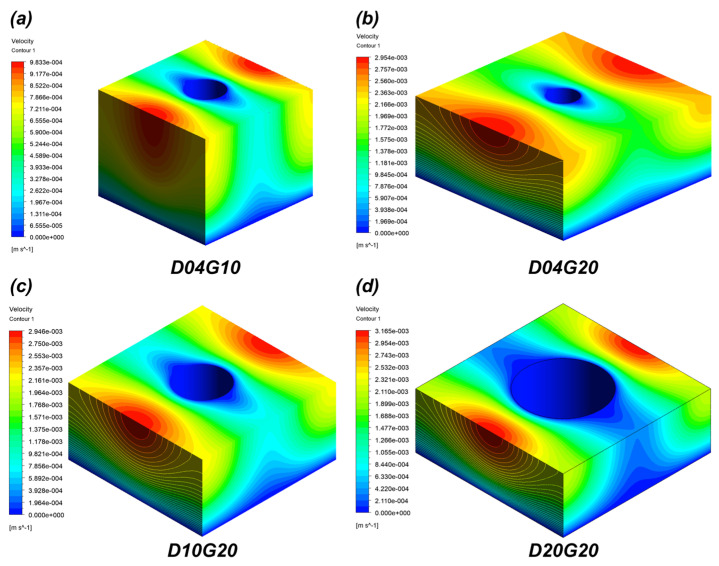
Velocity distribution for four samples at 20 °C obtained from CFD: (**a**) D04G10; (**b**) D04G20; (**c**) D10G20; (**d**) D20G20.

Table 2 presents the capillary pressure, mean velocity, viscous resistance, and wicking coefficient obtained from CFD simulations. The CFD results at 20 °C showed that, consistent with IR experiments, wicking performance improved as the pillar diameter (D) increased and the pillar gap (G) decreased. This is because the micropillar structures enhance surface roughness factor, thereby increasing capillary pressure and improving liquid supply capability. The simulation results indicate that at 20 °C, the capillary pressure of the sample with the highest roughness factor (D04G10, *r* = 2.51) was approximately 739% greater than that of the sample with the lowest roughness factor (D04G20, *r* = 1.51). Figure 9 shows the velocity distribution for each sample at 20 °C.

**Figure 10:**
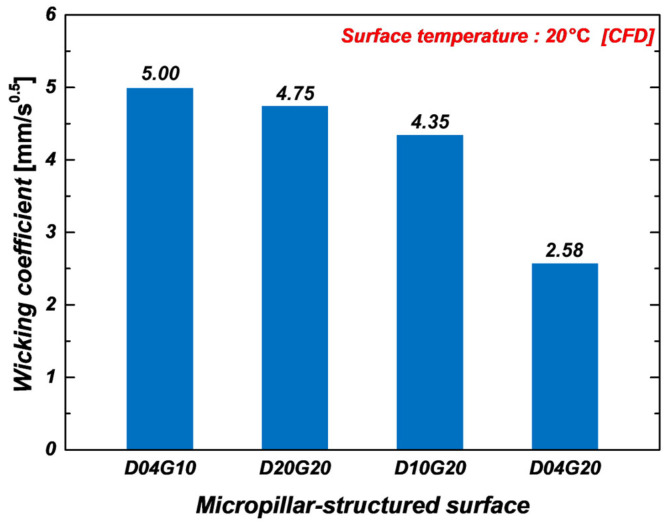
Wicking coefficients of micropillar samples at 20 °C measured using CFD simulation.

As discussed earlier, the wicking coefficients obtained from CFD simulations tended to be slightly higher than those from experiments. This discrepancy arises because experimental results reflect factors such as surface roughness, fabrication tolerances, and droplet evaporation, whereas CFD simulations assume an idealized environment. To address this, a fitting factor was applied. Figure 10 illustrates the wicking coefficients obtained from CFD simulations at 20 °C after incorporating a fitting factor of 0.468. The results show a strong correlation with experimental IR data, reducing discrepancies to within ±10%. This confirms that the CFD simulations achieve quantitative agreement with experimental measurements.

#### 3.2.2. CFD-Based Wicking Analysis Under Temperature Increase

To examine how wicking performance changes with increasing temperature, CFD simulations were conducted at 55 °C, 75 °C, and 95 °C. As shown in Table 2, an increase in temperature led to a decrease in viscous resistance (*K*) and an increase in mean velocity (*u*_mean_). Consequently, wicking coefficient also increased with temperature, a trend that aligns well with IR experimental results.

**Figure 11:**
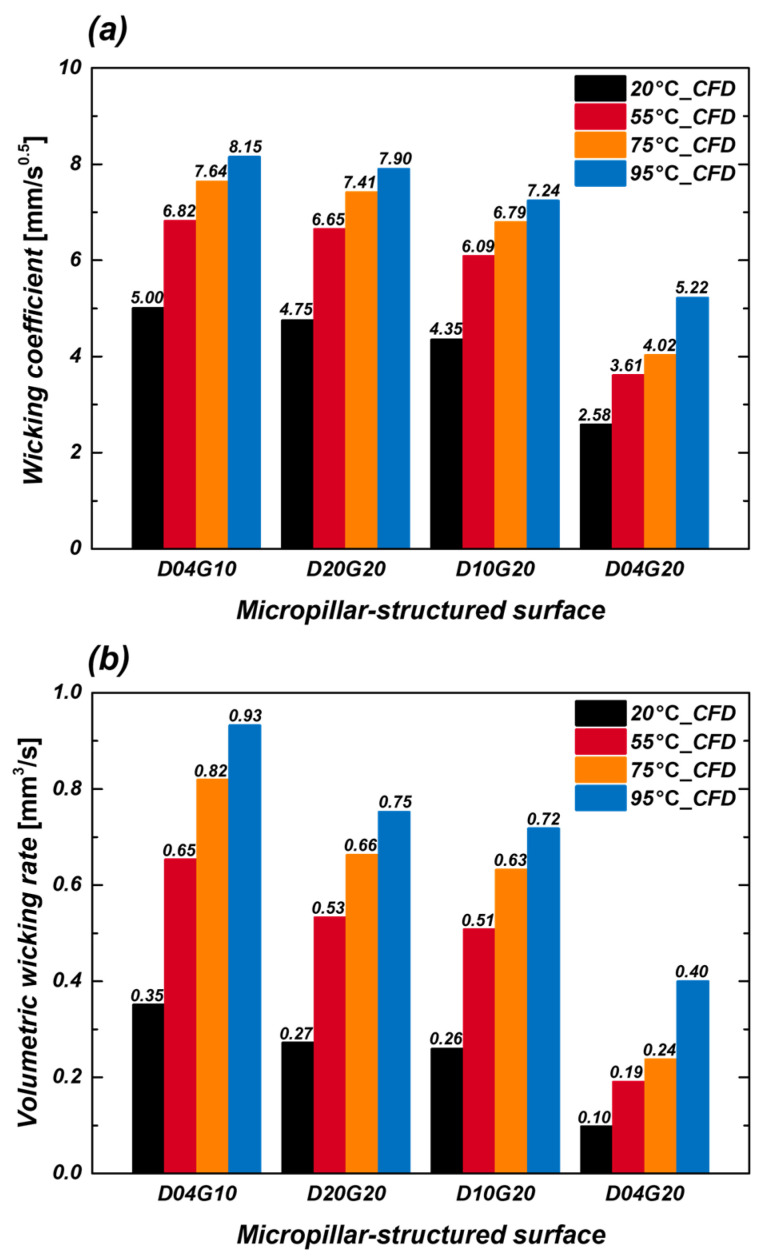
(**a**) Wicking coefficient; (**b**) Volumetric wicking rate of four samples at different temperatures obtained from CFD simulations.

Figure 11a shows the variation in wicking coefficient with temperature after applying the fitting factor. In all samples, the wicking coefficient increased with temperature, with the D04G10 sample exhibiting the highest increase. For instance, the wicking coefficient of the D04G10 sample increased from 5.00 mm/s^0.5^ at 20 °C to 8.15 mm/s^0.5^ at 95 °C, representing an approximately 63% increase. This improvement in wicking performance is attributed to the reduction in viscous resistance as temperature rises, while capillary pressure remains relatively high, facilitating fluid transport. The change in capillary pressure also plays a role in enhancing wicking performance, particularly in samples with high surface roughness factor (D04G10). Figure 11b presents the volumetric wicking rate as a function of temperature. Unlike wicking length alone, the volumetric wicking rate provides a more comprehensive evaluation of liquid supply efficiency, which is a crucial factor in optimizing fluid delivery in boiling heat transfer systems. The volumetric wicking rate ($\dot{V}$) was calculated using the following Equation (6):

(6) $$\dot{V}=W^{2}H(1-\varphi )$$

where H represents the pillar height. Similar to the wicking coefficient, the volumetric wicking rate increased with temperature. Specifically, the volumetric wicking rate of the D04G10 sample increased from 0.35 mm^3^/s^0.5^ at 20 °C to 0.93 mm^3^/s^0.5^ at 95 °C, showing an approximately 166% increase.

Through this study, it was confirmed that wicking performance improves with increasing temperature, as demonstrated by both experiments and CFD simulations. Furthermore, the IR visualization results exhibited trends consistent with those obtained from CFD simulations.

### 3.3. Comparison of Wicking Performance Between Experiments and CFD

In this study, wicking performance was measured under various temperature conditions using IR visualization experiments, and the results were compared with CFD simulations. The wicking coefficient and volumetric wicking rate were derived from IR experiments on micropillar structures, and discrepancies between these results and CFD simulations under identical conditions were analyzed to verify the reliability of the numerical model.

**Figure 12:**
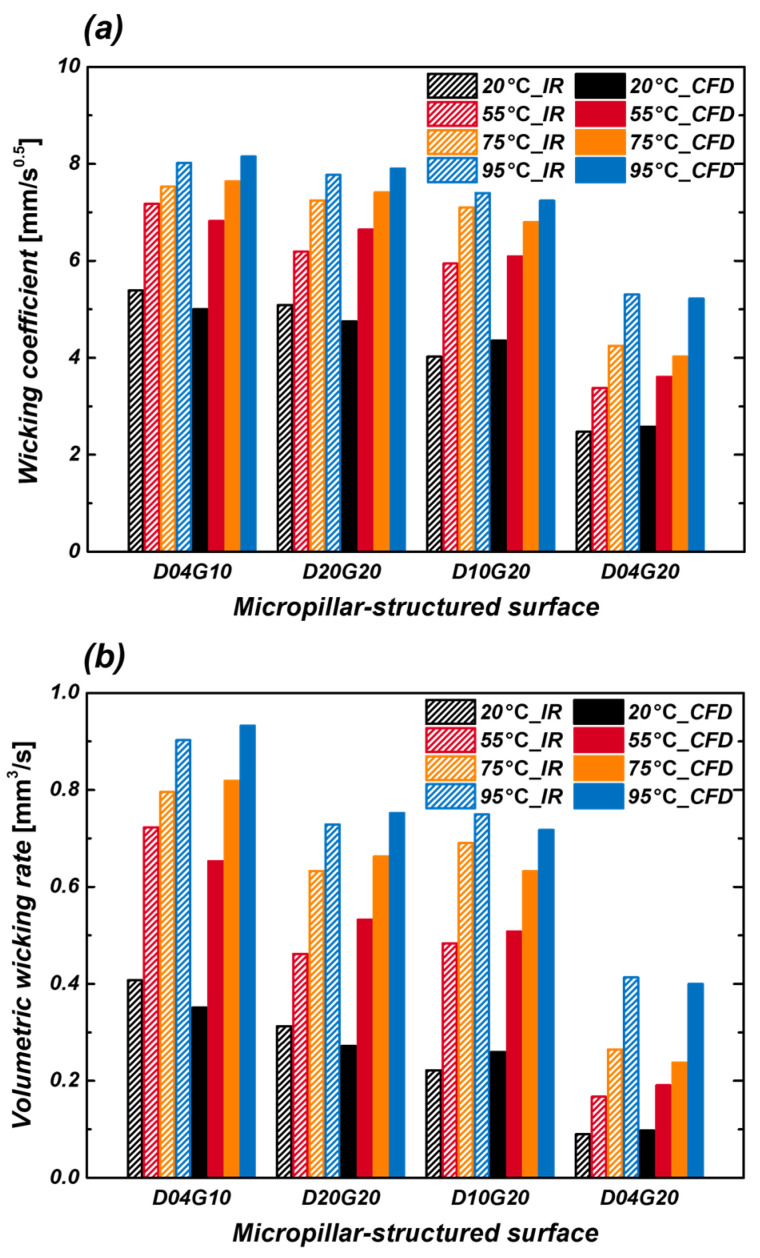
Comparison of (**a**) wicking coefficient and (**b**) volumetric wicking rate obtained from IR experiments with CFD simulation results that include a fitting factor.

**Figure 13:**
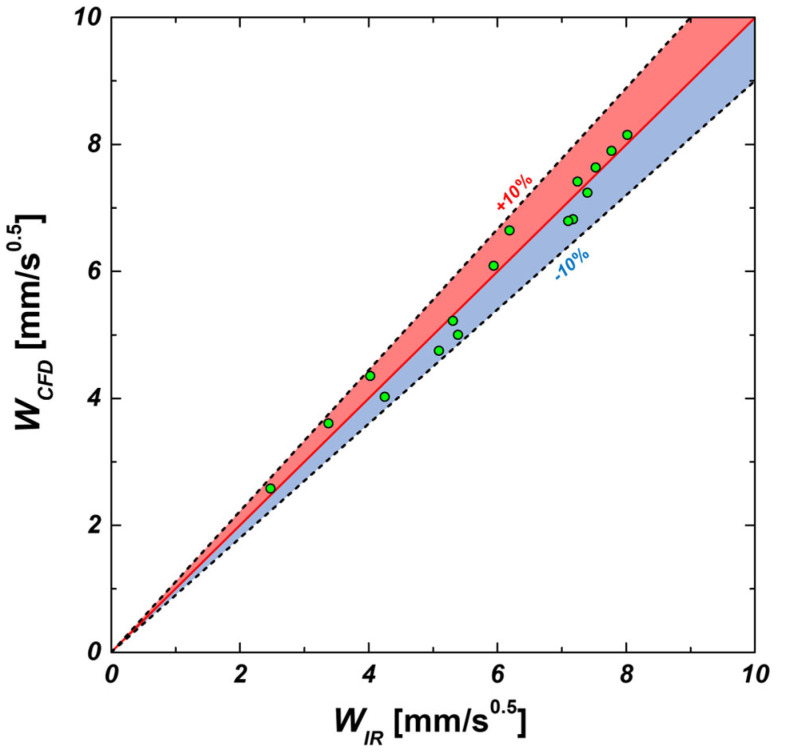
The error between wicking coefficients obtained from IR experiments and the results of CFD simulations that include a fitting factor.

Figure 12a,b present graphs comparing the wicking coefficient and volumetric wicking rate obtained from IR experiments with CFD simulation results that include a fitting factor, respectively. Overall, the wicking coefficient increased as temperature and surface roughness factor (increased pillar diameter and reduced gap) increased. This can be attributed to the decrease in viscous resistance and the relatively greater influence of capillary pressure at higher temperatures, which facilitates fluid transport. As the temperature rises, fluid viscosity decreases significantly, leading to a substantial reduction in viscous resistance. While capillary pressure also decreases slightly, the extent of this change is relatively small. Consequently, at higher temperatures, the influence of viscous resistance as a hindrance to fluid transport diminishes, making capillary pressure the dominant driving force. This explains why wicking performance improves as temperature increases. Figure 13 illustrates the error between the wicking coefficients obtained from IR experiments and the results of CFD simulations that include a fitting factor. The results show that experimental and CFD data maintain an error margin within ±10%, indicating the high reliability of the CFD model. Discrepancies between experimental and CFD results may stem from factors such as minor fabrication tolerances, droplet evaporation effects, or the resolution limitations of measurement equipment, which were not fully considered in the simulations. However, given that the error margin remains within 10%, the CFD model developed in this study is considered accurate enough to quantitatively reflect experimental results.

In conclusion, the CFD simulation results demonstrated strong correlation with IR experimental data, confirming that the developed numerical model can serve as a reliable tool for predicting wicking performance based on temperature and micropillar structure variations. Moving forward, additional CFD studies will be conducted on a broader range of micropillar design parameters (D02, D15, G05, G40, etc.), enabling a more comprehensive prediction of wicking performance. Future CFD simulations will also extend to different sample conditions (D02, D15, G05, G40, H20, etc.). These findings are expected to provide foundational data for the development of a new CHF correlation that accounts for fluid supply performance at elevated temperatures in boiling heat transfer systems.

## 4. Conclusions

This study experimentally measured the wicking performance of micropillar structures and quantitatively analyzed it using CFD simulations. IR visualization was employed to capture wicking phenomena under various temperature conditions, and the wicking coefficient was derived. The reliability of the numerical model was validated by comparing the CFD simulation results with experimental data. The experimental results showed that at room temperature (20 °C), the wicking coefficient increased as the pillar diameter (D) increased and the pillar gap (G) decreased. Specifically, the wicking coefficient of the sample with the highest roughness factor (D04G10) increased by 117% compared to the sample with the lowest roughness factor (D04G20). This can be attributed to the enhanced capillary pressure resulting from increased surface roughness factor in the micropillar structure, which subsequently improves fluid supply capability. Additionally, for samples with the same roughness factor, the wicking coefficient exhibited an increasing trend with rising temperature. In particular, the wicking coefficient of the D04G10 sample, which had the highest roughness factor, increased from 5.39 mm/s^0.5^ at 20 °C to 8.02 mm/s^0.5^ at 95 °C, reflecting a 49% rise. This trend can be explained by the significant reduction in viscous resistance due to decreased fluid viscosity, while the decrease in capillary pressure caused by reduced surface tension was relatively minor. As a result, the wicking coefficient increased with temperature, facilitating fluid transport. However, at higher temperatures above 95 °C, the increase in the wicking coefficient became less pronounced. This could be attributed to various factors, including droplet evaporation, measurement uncertainties, and the anisotropic nature of wicking. CFD simulations were conducted to predict wicking performance under temperature variations, and the CFD results were calibrated to align with experimental data. A comparison between experiments and simulations showed a strong agreement within an error margin of ±10%, confirming that the CFD model is a reliable tool for quantitatively predicting wicking performance. The data obtained in this study serve as essential reference material for developing a new CHF correlation that incorporates wicking performance near boiling temperatures. Furthermore, these findings are expected to contribute to the development of CHF prediction models and play a crucial role in next-generation cooling technologies based on capillary-driven flow, including nuclear reactor core cooling, computer chip cooling, and electric vehicle battery thermal management.

## Figures and Tables

**Table 1:** Detailed specifications of micropillar samples.

	Geometric Variables					CA	CCA
Sample	D(μm)	G(μm)	H(μm)	r(-)	φ(-)	θ (°)	$\theta_{c}({}^{\circ})$
D04G10	4	10	15	2.51	0.064	6.11	67.5
D04G20	4	20	15	1.51	0.022	4.66	49.0
D10G20	10	20	15	1.82	0.087	5.39	58.3
D20G20	20	20	15	1.93	0.196	8.11	62.3

**Table 2:** The wicking characteristics obtained from CFD simulations.

Temp. (°C)	Sample	$P_{cap}\;(N/m^{2})$	$u_{mean}\;\left(\times 10^{-3}\;m/s\right)$	$K\;\left(\times 10^{6}\;Pa\cdot s/m^{2}\right)$	$W\;\left(mm/s^{0.5}\right)$
20 °C	D04G10	4687.96	0.61	82.11	10.69
	D04G20	559.07	1.36	36.84	5.51
	D10G20	2066.46	1.05	47.77	9.30
	D20G20	3141.22	0.82	60.98	10.15
55 °C	D04G10	4262.95	1.25	40.14	14.57
	D04G20	508.39	2.92	17.12	7.71
	D10G20	1879.11	2.25	22.19	13.01
	D20G20	2856.44	1.77	28.33	14.20
75 °C	D04G10	4031.13	1.65	30.27	16.32
	D04G20	480.74	3.84	13.00	8.60
	D10G20	1776.93	2.97	16.86	14.52
	D20G20	2701.10	2.32	21.52	15.84
95 °C	D04G10	3850.82	1.97	25.40	17.41
	D04G20	459.24	6.78	7.38	11.16
	D10G20	1697.45	3.52	14.19	15.05
	D20G20	2580.29	2.76	18.11	16.88

## Data Availability

The original contributions presented in the study are included in the article, further inquiries can be directed to the corresponding author.
